# Improved differentiation of cavernous malformation and acute intraparenchymal hemorrhage on CT using an AI algorithm

**DOI:** 10.1038/s41598-024-61960-0

**Published:** 2024-05-23

**Authors:** Jung Youn Kim, Hye Jeong Choi, Sang Heum Kim, Hwangseon Ju

**Affiliations:** Department of Radiology, CHA Bundang Medical Center, CHA University, 59 Yatap-Ro, Bundang, Seongnam, Gyeonggi-Do 13496 Republic of Korea

**Keywords:** Diseases, Medical research, Neurology

## Abstract

This study aimed to evaluate the utility of an artificial intelligence (AI) algorithm in differentiating between cerebral cavernous malformation (CCM) and acute intraparenchymal hemorrhage (AIH) on brain computed tomography (CT). A retrospective, multireader, randomized study was conducted to validate the performance of an AI algorithm in differentiating AIH from CCM on brain CT. CT images of CM and AIH (< 3 cm) were identified from the database. Six blinded reviewers, including two neuroradiologists, two radiology residents, and two emergency department physicians, evaluated CT images from 288 patients (CCM, n = 173; AIH, n = 115) with and without AI assistance, comparing diagnostic performance. Brain CT interpretation with AI assistance resulted in significantly higher diagnostic accuracy than without (86.92% vs. 79.86%, *p* < 0.001). Radiology residents and emergency department physicians showed significantly improved accuracy of CT interpretation with AI assistance than without (84.21% vs. 75.35%, 80.73% vs. 72.57%; respectively, *p* < 0.05). Neuroradiologists showed a trend of higher accuracy with AI assistance in the interpretation but lacked statistical significance (95.83% vs. 91.67%, *p* = 0.56). The use of an AI algorithm can enhance the differentiation of AIH from CCM in brain CT interpretation, particularly for nonexperts in neuroradiology.

## Introduction

Cerebrovascular diseases remain an important concern in the clinical setting, with a range of pathologies requiring precise diagnostic differentiation for appropriate patient management. Cerebral cavernous malformation (CCM) and small acute intraparenchymal hemorrhage (AIH) represent two distinct entities that require accurate differentiation. However, their differentiation can be particularly challenging on brain computed tomography (CT) due to the similarity of imaging findings on initial presentation, especially in the setting of the emergency department^1–6^.

CCM, a vascular anomaly, is characterized by dilated sinusoidal vascular channels lined with a single layer of endothelium lacking the full complement of mature vessel wall components, with an estimated prevalence between 0.4 and 0.8% of the population^7–10^. Although CCMs may complicate with acute hemorrhage, most cases are incidentally detected on imaging studies and no further intervention or treatment is indicated unless they become complicated or symptomatic^7,11^. On the other hand, AIH, often resulting from hypertensive vasculopathy, necessitates prompt recognition for timely therapeutic interventions^12,13^. The accurate differentiation between CCM and AIH is important due to their distinct clinical implications and therapeutic strategies^7,12,13^. While CCM patients typically warrant surveillance and conservative management, AIH cases necessitate intervention to prevent potential complications and reduce morbidity and mortality^7,13^.

Neuroimaging plays a central role in the diagnostic pathway of these conditions^7,10,14^. However, despite the clinical importance, differentiation can be challenging even after brain CT evaluation, when lesions are small^1–6^. CCMs appear as focal areas of hyperdensity on CT, often attributed to calcium deposition or varying stages of blood content, and AIHs also commonly appear as uniform and smooth hyperdense lesions on CT due to the extravasation of blood content into the parenchyma^2,3,11,14–17^. The similarity in their initial radiological appearance on CT, particularly when lesions are small, can pose challenges in precise differentiation, even for experienced clinicians, commonly necessitating follow-up imaging or brain magnetic resonance imaging (MRI)^10,11,18,19^.

There has been an explosive increase in interest in artificial intelligence (AI) algorithms within recent clinical research, which has led to a rapid surge in their applications, including the detection and validation of AIH on brain CT scans^20–27^. However, to our knowledge, there has been no study differentiating CCM and AIH using an AI algorithm on brain CT images.

In this context, our study was designed to investigate the potential utility of an AI algorithm in distinguishing between CCM and AIH on brain CT images. In addition, we also assess how physician experience impacts diagnostic accuracy when utilizing AI assistance.

## Materials and methods

In this retrospective, multireader, randomized study, we aimed to differentiate between CCM and AIH on brain CT images using a commercially available deep learning-based AI algorithm (Medical Insight + Brain Hemorrhage, SK Inc. C&C, Seongnam, Republic of Korea)^20^. This study was conducted in accordance with the principles of the Declaration of Helsinki and was approved by the institutional review board (IRB) of CHA Bundang Medical Center (IRB No. 2023-10-009-003). The requirement for informed consent was waived due to the retrospective nature of the study. All study methods were performed in accordance with relevant guidelines and regulations.

We searched our tertiary hospital’s radiology information system and picture archiving and communication system (PACS) crawler using keywords related to CCM (cavernous malformation(s), cavernous angioma(s), cavernous hemangioma(s), cavernoma(s)) to retrieve all noncontrast brain CT examinations conducted between January 2001 and October 2022. Additionally, patients diagnosed with AIH through the International Classification of Diseases, 10th revision, medical diagnostic codes of “nontraumatic intracerebral hemorrhage” in our hospital between January 2019 and December 2021 were searched for.

Two experienced neuroradiologists, each with 11 and 13 years of neuroimaging expertise, jointly reviewed consecutive imaging studies of all identified brain CT cases and established the gold standard diagnosis through consensus. For the diagnosis of CCM, stability over a period of more than 1 month on CT or MRI, or the presence of a typical popcorn-ball appearance with a blooming effect on T2* images without signs of acute hemorrhage or perilesional edema on MRI^18,28–31^, which is performed within 1 month of the target CT images, was needed.

**Figure 1:**
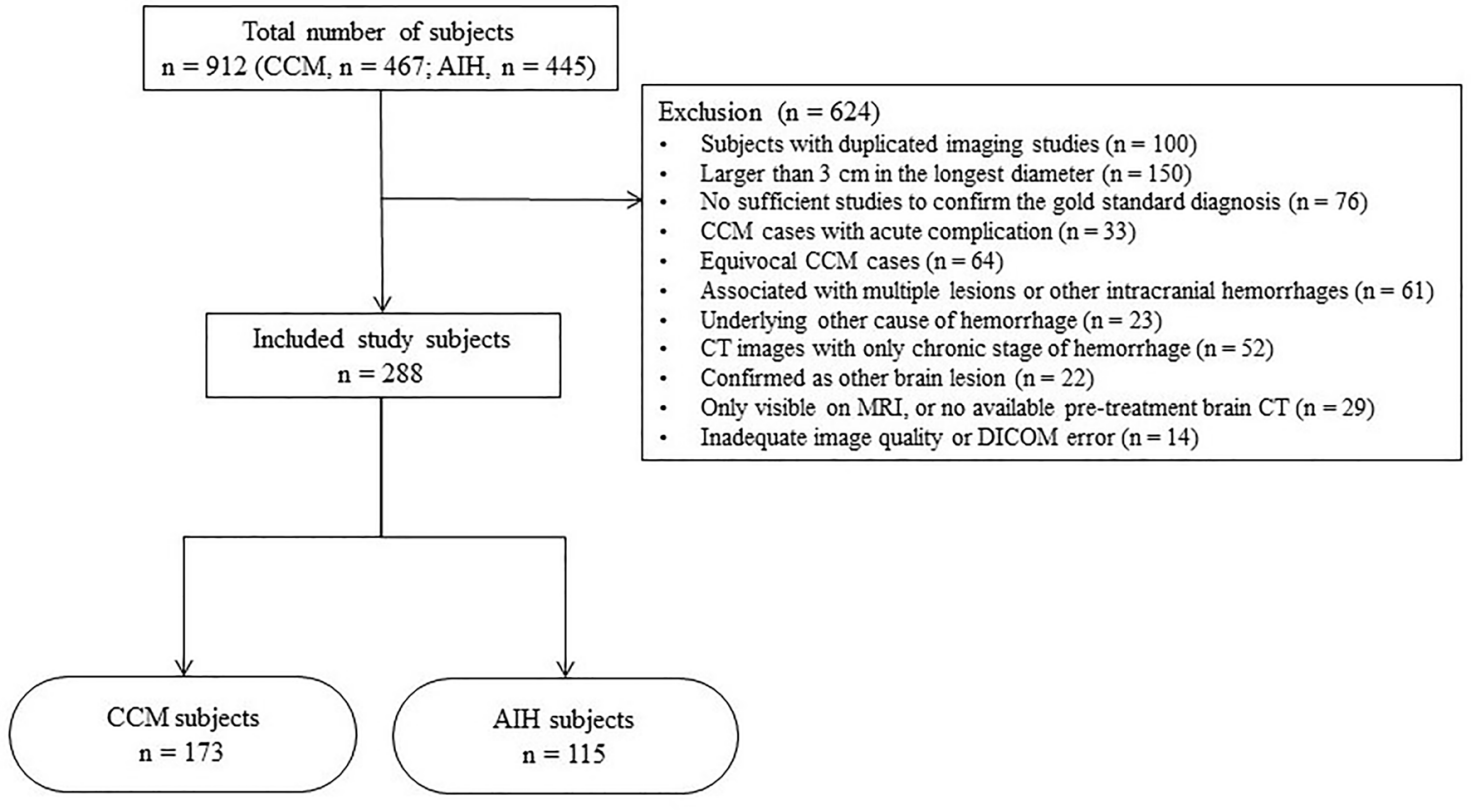
Flow diagram of the included patients. CCM, cerebral cavernous malformation; AIH, acute intraparenchymal hemorrhage; CT, computed tomography.

Of a total 912 patients (CCM, n = 467; AIH, n = 445), 624 patients (CCM, n = 294; AIH, n = 330) were excluded for the following reasons: (a) patients with duplicated imaging studies (n = 100), and in case of duplicated patients the initial available brain CT was selected for the image analysis; (b) AIH and CCM cases larger than 3 cm in the longest diameter (n = 150), as previous study have indicated a greater risk of hemorrhage in CCM cases larger than 3 cm^32^; (c) suspected CCM cases with no sufficient follow-up or work-up studies to confirm the gold standard diagnosis (n = 76); (d) CCM cases with acute complication confirmed by recent hemorrhagic signal/density or perilesional edema that showed changes at follow-up imaging (n = 33); (e) equivocal CCM lesions, that could not be differentiated from calcified granuloma (n = 64); (f) associated with multiple lesions, or other intracranial hemorrhages such as intraventricular, subarachnoid, subdural hemorrhages (n = 61); (g) AIH cases with other underlying cause of hemorrhage, including intracranial tumor, cerebral infarction, Moyamoya disease, or recent trauma history (n = 23); (h) searched cases with only CT images of chronic stage of hemorrhage (n = 52); (i) confirmed as other brain lesion other than AIH or CCM, such as calcified meningioma, cavernous sinus hemangioma, arteriovenous malformation, nonketotic hyperglycemic hemiballismus, or else (n = 22); (j) cases which are only visible on MRI, or no available pretreatment brain CT for image analysis (n = 29); and (k) unavailability of image analysis due to inadequate image quality or DICOM error (n = 14). A flow diagram of the included study patients is shown in Fig. 1.

**Figure 2:**
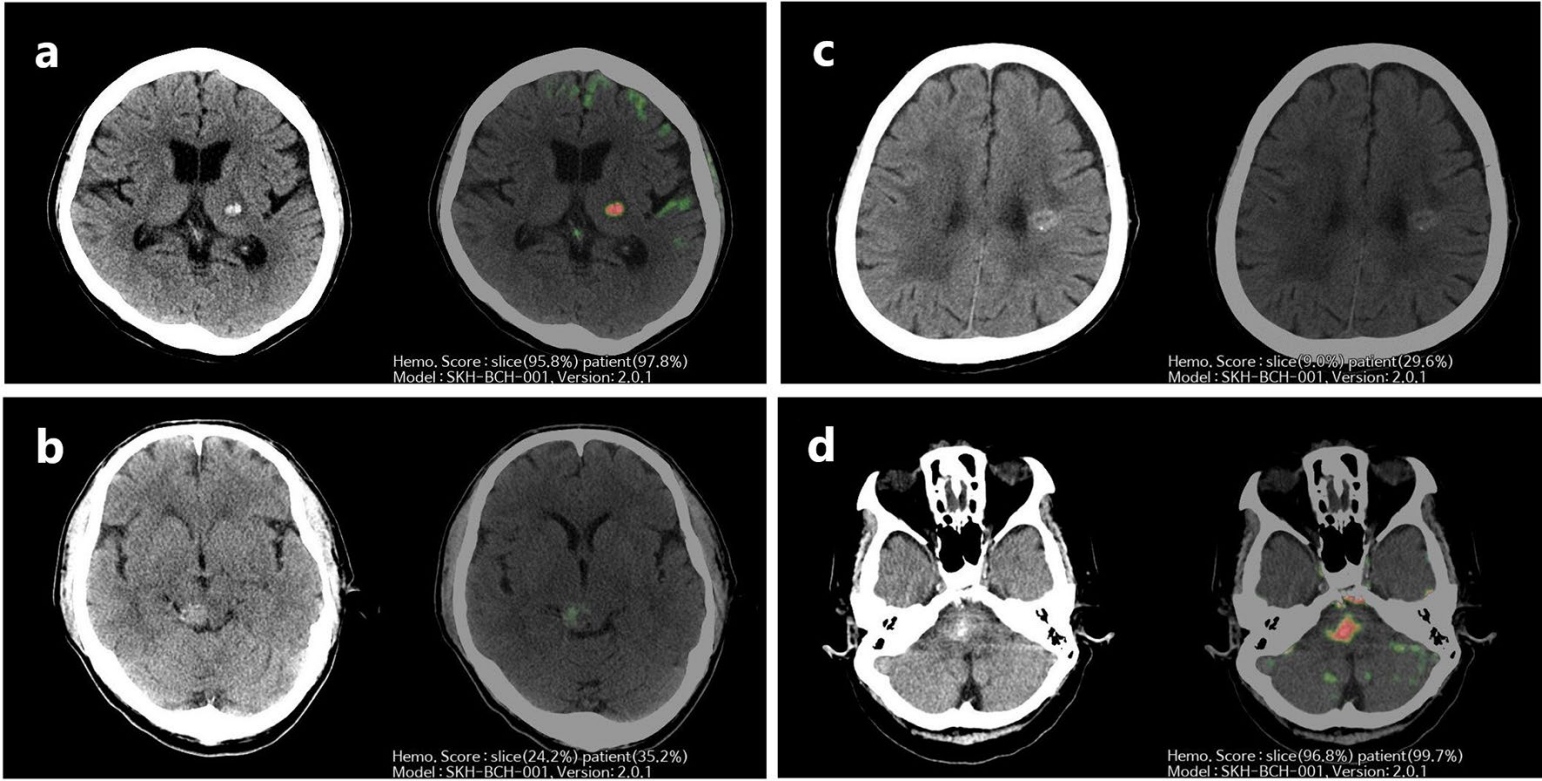
Representative images of the AI algorithm for differentiating CCM and AIH. AI, artificial intelligence; CCM, cerebral cavernous malformation; AIH, acute intraparenchymal hemorrhage; CT, computed tomography. (**a**) Brain CT shows AIH in the left thalamus. AI-assisted brain CT analysis provided probability scores of hemorrhage in a slicewise (95.8%) and patientwise (97.8%) manner. All six reviewers correctly interpreted the images as AIH for both AI-unassisted and AI-assisted interpretations. **(b)** Brain CT shows a CCM on the right side of the midbrain. AI-assisted brain CT analysis provided a probability score of hemorrhage in a slicewise (24.2%) and patientwise (35.2%) manner. One radiology resident and one emergency department physician incorrectly interpreted the images as AIH in the AI-unassisted brain CT. However, with the use of AI assistance, all reveiwers correctly diagnosed the images as CCM. (**c**) Brain CT shows a CCM in the left corona radiata. AI-assisted brain CT analysis generated probability scores of hemorrhage in a slicewise (9.0%) and patientwise (29.6%) manner. All six reviewers accurately identified the images as CCM for both AI-unassisted and AI-assisted interpretations. (**d**) Brain CT shows AIH in the pons. AI-assisted brain CT analysis generated a probability score of hemorrhage in a slicewise (96.8%) and patientwise (99.7%) manner. All neuroradiologists and emergency department physicians erroneously interpreted the images as CCM in the AI-unassisted brain CT. Nevertheless, with the use of AI assistance, all reviewers accurately diagnosed the images as AIH.

Therefore, a total of 288 complete CT images (CCM, n = 173; AIH, n = 115) that satisfied the criteria for image analysis were enrolled in the study. The CT images were processed using the AI algorithm, generating heatmaps and probability scores through slicewise and patientwise analysis, highlighting possible hemorrhage locations. These results were displayed alongside the original CT images on the PACS viewer (Fig. 2).

A panel of six reviewers from three different subgroups with equal numbers (two emergency department physicians with 2–7 years of experience in that role, two radiology residents with 2–3 years of radiology, and two experienced neuroradiologists with 11–13 years of experience in neuroradiology) from two institutions in South Korea (CHA Bundang Medical Center and CHA Gumi Medical Center) participated in the assessment. In this retrospective, multireader, randomized study, the first assessment was conducted from the dataset with only CT images and the second assessment was conducted from the dataset composed of CT images and corresponding AI-assisted CT images of the included patients. The second assessment was performed after a 4-week of washout period following the first assessment. Additionally, the order of dataset images for assessments was randomized for each session. Each reviewer independently reviewed the CT images from the 288 patients with and without AI assistance for differentiation of AIH and CCM. Reviewers were blinded to the gold-standard diagnoses, proportions of AIH and CCM in the dataset, and patients’ clinical data.

Clinical data of the patients, including age, sex, history of hypertension and diabetes, smoking history, and blood pressure at presentation, were collected for analysis. Two neuroradiologists manually measured the size of the AIH or CCM on axial CT images from the longest diameter (size 1) and corresponding perpendicular diameter (size 2). Lesion locations were categorized according to specific brain regions.

The baseline characteristics between subjects with CCM and AIH were compared using the χ^2^ test for categorical variables and Student’s *t* test or the Mann‒Whitney test for continuous variables, as appropriate. The sensitivity, specificity, and accuracy of AI-assisted and AI-unassisted assessments were compared using the χ^2^ test. The diagnostic performance of AI-assisted and AI-unassisted assessments across reviewer subgroups was evaluated using the confusion matrix and generalized estimating equation (GEE) method. All analyses were conducted using R studio version 4.2.3 (RStudio, Boston, MA, USA) and SAS statistical software version 9.4 (SAS Instituted, Cary, NC, USA). A *p* value of less than 0.05 was considered statistically significant.

## Results

The characteristics of the 288 study participants are shown in Table 1. The mean age of patients with CCM was 53.49 ± 16.27 years, whereas patients with AIH had a mean age of 61.25 ± 14.15 years. Male patients constituted 57.29% of the total cohort, with a higher proportion among the AIH group (62.61%). The prevalence of hypertension and smoking history significantly differed between the CCM and AIH groups (30.06% vs. 50.43%, 16.76% vs. 29.57%, respectively, *p* < 0.05) and significant differences in systolic and diastolic blood pressures were also observed. Medical history of diabetes were comparable between the two groups. There was a notable difference in the predominant presenting symptom between CCM and AIH patients. Among CCM patients, the predominant presenting symptom was headache, followed by dizziness and seizure or syncope. In contrast, the most common presenting symptom among AIH patients was motor weakness followed by dizziness and dysarthria.

**Table 1:** Patient and clinical characteristics.

	Total (N = 288)	CCM (N = 173)	AIH (N = 115)	*P* value
Age, years [SD]	56.59 [15.90]	53.49 [16.27]	61.25 [14.15]	< 0.001
Male, n (%)	165 (57.29)	93 (53.76)	72 (62.61)	0.172
Hypertension, n (%)	110 (38.19)	52 (30.06)	58 (50.43)	0.001
DM, n (%)	50 (17.36)	25 (14.45)	25 (21.74)	0.15
Smoking, n (%)	63 (21.88)	29 (16.76)	34 (29.57)	0.015
Systolic BP, mmHg [SD]	141.9 [32.27]	131.45 [24.51]	158.16 [36.00]	< 0.001
Diastolic BP, mmHg [SD]	83.58 [18.64]	80.36 [15.76]	88.55 [21.53]	0.001
Presenting symptom, n (%)				
Headache	68 (23.61)	57 (32.95)	11 (9.57)	< 0.001
Dizziness	54 (18.75)	40 (23.12)	14 (12.17)	
Motor weakness	37 (12.85)	4 (1.39)	33 (28.70)	
Dysarthria	18 (6.25)	5 (2.89)	13 (11.30)	
Mental change	11 (3.82)	2 (1.16)	9 (7.83)	
Seizure	10 (3.47)	7 (4.05)	3 (2.61)	
Syncope	8 (2.78)	7 (4.05)	1 (0.85)	
Others or nonspecific	82 (28.47)	51 (29.48)	31 (29.96)	
Size1^a^, cm [SD]	14.57 [6.79]	11.31 [4.57]	19.49 [6.62]	< 0.001
Size2^b^, cm [SD]	10.74 [5.18]	8.55 [3.46]	14.0 [5.60]	< 0.001
Location, N (%)				
Lobar	134 (46.53)	119 (68.79)	15 (13.04)	< 0.001
Basal ganglia	48 (16.67)	6 (3.47)	42 (36.52)	
Thalamus	41 (14.24)	6 (3.47)	35 (30.43)	
Cerebellum	30 (10.42)	23 (13.29)	7 (6.09)	
Pons	24 (8.33)	10 (5.78)	14 (12.17)	
Midbrain	5 (1.74)	3 (1.73)	2 (1.74)	
Corpus callosum	4 (1.39)	4 (2.31)	0 (0)	
Medulla	2 (0.69)	2 (1.16)	0 (0)	
Slicewise AI, % [SD]	63.71 [38.79]	43.82 [37.17]	93.62 [14.15]	< 0.001
Patientwise AI, % [SD]	69.44 [35.08]	52.25 [35.21]	95.31 [10.22]	< 0.001

CCM, cerebral cavernous malformation; AIH, acute intraparenchymal hemorrhage; n, patient number; SD, standard deviation; DM, diabetes mellitus; BP, blood pressure; AI, artificial intelligence.

^a^ Longest diameter of the lesion measured on axial CT images.

^b^ Diameter of the lesion perpendicular to size 1 measured on axial CT images.

Regarding the target lesion, the sizes (size 1 and size 2) were slightly larger in the AIH groups (14.57 mm and 10.74 mm, respectively) than in the CCM groups (11.31 mm and 8.55 mm, respectively) with statistical significance. The distribution of lesion locations was also significantly different. The majority of CCM lesions were found in the lobar regions (68.79%), whereas AIH lesions were predominantly located in the basal ganglia (36.52%) and thalamus (30.43%).

The AI standalone performance for differentiating between CCM and AIH achieved a diagnostic accuracy of 87.5% and 85.76%, with a sensitivity of 97.1% and 94.22%, and a specificity of 73.04% and 73.04% in slicewise and patientwise analyses, respectively.

The diagnostic performance with the use of AI assistance and without AI assistance among the three subgroups of reviewers was compared (Table 2). In the total group of reviewers, AI-assisted assessment significantly improved accuracy (86.92% vs. 79.86%), sensitivity (81.21%, 71.39%), and specificity (95.51% vs. 92.61%) compared to AI-unassisted assessment. Overall diagnostic accuracy varied among the three subgroups of reviewers, with reviewers in neuroradiologist subgroup demonstrating the highest accuracy, followed by those in the radiology resident and emergency department physician subgroups. When comparing AI-assisted and AI-unassisted assessments, statistically significant improvements in diagnostic accuracy were observed for radiology residents (84.21% vs. 75.35%, with a difference of 8.86%, *p* < 0.05) and emergency department physicians (80.73% vs. 72.57%, with a difference of 8.16%, *p* < 0.05). However, among neuroradiologists, although the AI-assisted differentiation of CCM and AIH showed a higher diagnostic accuracy than unassisted assessment, this difference did not reach statistical significance (95.83% vs. 91.67%, with a difference of 4.16%, *p* = 0.56).

**Table 2:** Diagnostic performances of each subgroup after conducting AI-unassisted or AI-assisted evaluation for differentiating CCM and AIH.

Reviewers	AI-unassisted	AI-assisted	*P* value
Total reviewers (%) (95% confidence interval)			
Accuracy	79.86 (75.03, 84.05)	86.92 (82.77, 90.28)	< 0.001
Sensitivity	71.39 (68.56, 74.05)	81.21 (78.72, 83.47)	
Specificity	92.61 (90.41, 94.33)	95.51 (93.69, 96.81)	
Emergency department physicians			
Accuracy	72.57 (67.29, 77.38)	80.73 (75.86, 84.96)	0.006
Sensitivity	59.53 (54.29, 64.58)	70.23 (65.21, 74.81)	
Specificity	92.17 (87.97, 94.99)	96.52 (93.29, 98.22)	
Radiology residents			
Accuracy	75.35 (69.95, 80.21)	84.21 (79.63, 88.04)	< 0.001
Sensitivity	63.01 (57.80, 67.92)	77.17 (72.46, 81.28)	
Specificity	93.91 (90.04, 96.33)	94.78 (91.10, 96.99)	
Neuroradiologists			
Accuracy	91.67 (87.88, 94.57)	95.83 (92.83, 97.83)	0.56
Sensitivity	91.62 (88.22, 94.10)	96.24 (93.68, 97.79)	
Specificity	91.74 (87.46, 94.65)	95.22 (91.64, 97.31)	

AI, artificial intelligence; CCM, cerebral cavernous malformation; AIH, acute intraparenchymal hemorrhage.

## Discussion

The findings of our study highlight the substantial potential of AI algorithms in effectively differentiating between cases of CCM and AIH. The observed enhancement in diagnostic performance through AI assistance underscores its pivotal role in facilitating clinicians’ prompt and precise decision-making, particularly for nonexpert neuroradiologists.

Distinguishing between CCM and AIH at the initial patient presentation can be challenging, but clinically crucial due to the divergent clinical management pathways these conditions entail ^7,12,13,33^. The distinctive characteristics and implications of these two pathologies necessitate precise differentiation for appropriate treatment or follow up planning. The integration of AI algorithms into clinical practice has the potential to expedite the diagnostic process, thus contributing to more timely patient care and reducing unnecessary examinations. While previous studies have reported the benefits of utilizing AI algorithms in detecting or classifying intracranial hemorrhages ^20–25^, our study represents a novel contribution as the first report to utilize AI algorithm to differentiate between CCM and AIH on brain CT images.

In the reviewer assessments, which adopted a retrospective, multireader, randomized study design, the AI-assisted assessment exhibited significantly higher diagnostic accuracy in distinguishing between CCM and AIH compared to the AI-unassisted assessment. The disparities in diagnostic accuracy observed across different reviewer subgroups are of considerable interest. The neuroradiologist subgroup, representing specialized expertise, exhibited the highest accuracy. Even among experts, the incremental improvement in accuracy with AI assistance, while not reaching statistical significance, emphasizes the complementary role of AI in assisting highly skilled professionals. Furthermore, both radiology residents and emergency department physicians demonstrated statistically significant enhancements in diagnostic accuracy when utilizing AI-assistance. However, they did not surpass the diagnostic accuracy achieved by AI-unassisted neuroradiologists. Our study results are in concordance with those of previous research utilizing AI assistance in detecting intracranial hemorrhage, which showed enhanced diagnostic accuracy among nonexpert practitioners ^20^. These promising results suggest that AI has the potential to bridge the gap between expert and nonexpert physicians, thereby contributing to consistent and accurate diagnoses across a diverse spectrum of healthcare providers, especially in clinical settings where prompt interpretation from expert neuroradiologists is not readily accessible.

While our study demonstrates the beneficial role of AI assistance, its introduction into healthcare system necessitates careful consideration of its impact on skill acquisition and maintenance among medical trainees, as emphasized by Chassagnon et al. ^34^. Addressing this issue requires ongoing collaboration between medical educators, healthcare providers, and AI developers to optimize training programs and ensure the responsible integration of AI into clinical practice. This collaboration is essential for cultivating radiology experts and ensuring that AI complements and enhances the capabilities of healthcare professionals, rather than replacing their expertise.

There are several limitations of this study that should be noted. First, determination of CCM and AIH is challenging, particularly when the lesion is small and no further management is indicated. In routine clinical practice, the ground truth might remain elusive in such cases. To address this limitation, we implemented a rigorous approach and included patients whose management involved follow-up imaging to observe interval changes or MRI examinations for accurate diagnosis within a defined time frame. These assessments were carried out by consensus between two experienced neuroradiologists with a minimum of 11 and 13 years of radiology expertise, ensuring meticulous interpretation. Second, the relatively limited number of reviewers might have introduced a potential influence on the generalizability of our findings. The composition of reviewers in our study may not be fully representative of the entire subgroup, introducing a degree of uncertainty in the robustness of our conclusions. Moreover, the varying levels of experience among the reviewers could have impacted the study outcomes, introducing an additional layer of complexity. Third, there is a possibility that the first assessment by the reviewers influenced the interpretation of the second assessment. To mitigate this potential bias, the second assessment was conducted after a 4-week washout period, and the order of dataset images for assessments was randomly reorganized. Fourth, although we meticulously selected cases without other causes of AIH to eliminate secondary hemorrhages, the possibility of an underlying cause of hemorrhage that was not identified within our institute remains a potential limitation of this study. Fifth, the retrospective nature of our study and inherent demographic traits of the study population give rise to the possibility of inherent biases and limitations associated with data collection. Further investigations involving larger and more diverse cohorts of patients and reviewers would be valuable to validate and extend our findings. Nonetheless, this study represents the first attempt to apply an AI algorithm in distinguishing CCM and AIH using brain CT images while also comparing the diagnostic accuracy across different levels of physician experience.

In conclusion, our study provides compelling evidence of the substantial potential of AI algorithms in differentiating between CCM and AIH on brain CT images. The improved diagnostic performance achieved through AI assistance highlights its valuable role in aiding clinicians, particularly those lacking expertise in neuroradiology.

## Data Availability

The dataset analyzed during this study is not publicly available as it consists of patients’ sensitive personal information. The data are available from the Bundang CHA Medical Center upon reasonable request by qualified researchers trained in research with human subjects. Every request will be reviewed by the Institutional Review Board of Bundang CHA Medical Center, and researchers can access the data according to the approval conditions.
